# Hemodynamic Outcomes and Vasopressor Requirements with Remimazolam-Based Total Intravenous Anesthesia Versus Propofol–Sevoflurane Anesthesia in Robot-Assisted Direct-to-Implant Breast Reconstruction

**DOI:** 10.3390/medicina62101907

**Published:** 2026-10-02

**Authors:** Ji Young Kim, Na Young Kim, Hyun Joo Kim, Byongnam Jun, Joung Goo Cho, Minjeong Kim, Hyung Seok Park, Ju Hye Lee, Soo Jung Park

**Affiliations:** 1 Department of Anesthesiology and Pain Medicine, Anesthesia and Pain Research Institute, Yonsei University College of Medicine, Seoul 03722, Republic of Korea; amyjykim@yuhs.ac (J.Y.K.); knnyyy@yuhs.ac (N.Y.K.); bnjun@yuhs.ac (B.J.); 2 Department of Anesthesiology and Pain Medicine, Anesthesia and Pain Research Institute, Yonsei University College of Medicine, Gangnam Severance Hospital, 211 Eonju-ro, Gangnam-gu, Seoul 06273, Republic of Korea; jjollong@yuhs.ac; 3 Department of Anesthesiology and Pain Medicine, National Health Insurance Service Ilsan Hospital, Gyeonggi-do, Goyang-si 10444, Republic of Korea; anaper@nhimc.or.kr (J.G.C.); goto9035@yuhs.ac (M.K.); 4 Division of Breast Surgery, Department of Surgery, Yonsei University College of Medicine, Seoul 03722, Republic of Korea; imgenius@yuhs.ac; 5 Department of Obstetrics and Gynecology, College of Medicine, Ewha Womans University Mokdong Hospital, Seoul 07985, Republic of Korea; ogjuhye@gmail.com

**Keywords:** remimazolam, total intravenous anesthesia, sevoflurane, robot-assisted surgery, breast reconstruction, intraoperative hypotension, vasopressor

## Abstract

*Background and Objectives*: Robot-assisted direct-to-implant breast reconstruction involves procedural factors that may affect intraoperative hemodynamics, including prolonged surgery, semi-sitting positioning, and subcutaneous carbon dioxide insufflation. This study compared vasopressor requirements and hemodynamic profiles between remimazolam-based total intravenous anesthesia (TIVA) and propofol–sevoflurane anesthesia. *Materials and Methods*: This retrospective cohort study included 46 patients who underwent robot-assisted direct-to-implant breast reconstruction. Of these, 20 received propofol–sevoflurane anesthesia, and 26 received remimazolam-based TIVA. The primary outcome was total intraoperative norepinephrine consumption. Secondary outcomes included vasopressor infusion duration, time from induction to vasopressor initiation, incidence of hypotension, hemodynamic profiles analyzed using linear mixed-effects models, postoperative pain scores, and reconstructive outcomes. *Results*: Total norepinephrine consumption was significantly lower in the remimazolam group than in the propofol–sevoflurane group (median, 123.2 [interquartile range, 62.4–176.4] vs. 580.0 [338.8–790.4] µg; *p* < 0.001). The remimazolam group also had a shorter vasopressor infusion duration (67.7 ± 40.0 vs. 176.3 ± 66.4 min; *p* < 0.001), a longer vasopressor-free interval from induction (median, 140 vs. 25 min; log-rank *p* < 0.001), and a lower incidence of hypotension, defined as mean arterial pressure (MAP) < 65 mmHg (46.2% vs. 80.0%; *p* = 0.032). After adjustment for age, body mass index, and anesthesia time, remimazolam-based anesthesia remained associated with lower norepinephrine consumption (*B* = −515.4 µg; *p* < 0.001). MAP and heart rate (HR) showed no significant between-group differences over time, and neither the estimated duration nor time-weighted average (TWA) below MAP 65 mmHg differed significantly. *Conclusions*: In this retrospective cohort, a remimazolam-based TIVA strategy was associated with lower recorded intraoperative norepinephrine use than a propofol–sevoflurane strategy. No significant between-group differences in serial MAP or HR were detected. These associations warrant confirmation in prospective trials with standardized protocols.

## 1. Introduction

Robot-assisted nipple-sparing mastectomy with immediate direct-to-implant (DTI) reconstruction has emerged as a minimally invasive approach for selected patients with breast cancer, offering small remote incisions, preservation of the nipple–areolar complex, and single-stage reconstruction [1,2,3]. Compared with conventional mastectomy followed by staged tissue expansion, this technique is associated with favorable cosmetic outcomes and high patient satisfaction [2,4].

However, successful DTI reconstruction depends critically on adequate perfusion of the mastectomy skin flap because compromised perfusion may lead to skin necrosis, implant exposure, and reoperation [5,6]. Robot-assisted DTI breast reconstruction involves several procedural factors that may influence intraoperative hemodynamics, including prolonged operative duration, subcutaneous carbon dioxide (CO_2_) insufflation, and semi-sitting positioning with ipsilateral arm elevation [7].

Although propofol induction followed by sevoflurane maintenance is a well-established and widely used approach, its cardiovascular effects may be of particular concern in this setting. Propofol can cause dose-dependent myocardial depression and vasodilation, whereas sevoflurane may reduce systemic vascular resistance [8,9,10]. These effects may contribute to intraoperative hypotension or increased vasopressor requirements in susceptible patients.

Remimazolam is an ultrashort-acting benzodiazepine that undergoes organ-independent metabolism by tissue esterases, resulting in a predictable offset of action and limited hemodynamic depression at clinical doses [11,12]. Previous randomized trials and meta-analyses have reported that remimazolam-based total intravenous anesthesia (TIVA) is associated with lower rates of hypotension and reduced vasopressor requirements than propofol- or volatile-based anesthesia [13,14,15]. In addition, the availability of flumazenil as a specific antagonist allows pharmacological reversal of residual sedation [11].

Despite this pharmacological rationale, evidence regarding remimazolam-based TIVA in robot-assisted DTI breast reconstruction remains limited. Therefore, this retrospective study aimed to compare intraoperative vasopressor requirements and hemodynamic profiles between remimazolam-based TIVA and propofol–sevoflurane anesthesia in patients undergoing robot-assisted immediate DTI breast reconstruction.

## 2. Materials and Methods

### 2.1. Study Design and Ethical Approval

This was a retrospective, single-center, observational cohort study. The study protocol was approved by the Institutional Review Board (IRB No. 4-2025-1243), and the requirement for informed consent was waived because of the retrospective nature of the study. The study was conducted in accordance with the Declaration of Helsinki and reported in accordance with the Strengthening the Reporting of Observational Studies in Epidemiology guidelines.

### 2.2. Participants

We reviewed the electronic medical records of consecutive patients who underwent robot-assisted nipple-sparing mastectomy with immediate DTI breast reconstruction between November 2021 and September 2023. Patients were eligible if they were aged ≥ 20 years and classified as American Society of Anesthesiologists (ASA) physical status I–III. Patients were excluded if they received anesthetic regimens other than the two study strategies, such as desflurane-based anesthesia or mixed maintenance regimens combining volatile anesthetics and remimazolam; underwent concurrent surgery performed by another surgical team; required an intraoperative change in the surgical plan; were admitted to the intensive care unit postoperatively; or had substantial gaps in intraoperative arterial blood pressure recordings in the anesthesia record because of technical recording errors, as complete hemodynamic records were required to interpret vasopressor use and to derive the hypotension-related outcomes.

### 2.3. Anesthetic Management

Patients were categorized into two groups according to the anesthetic technique used. Because of the retrospective design, there was no formal allocation rule; the anesthetic strategy was determined by the attending anesthesiologist’s preference and by remimazolam availability during the early period after its introduction. In the propofol–sevoflurane group (Group S), anesthesia was induced with an intravenous (IV) bolus of propofol (approximately 1–1.5 mg/kg; Fresofol^®^, Fresenius Kabi, Graz, Austria) and concurrent sevoflurane (Sevofran™, Hana Pharm, Seoul, Republic of Korea) inhalation via a face mask, with anesthetic depth guided by continuous bispectral index (BIS; BIS Complete Monitoring System, Medtronic, Minneapolis, MN, USA) monitoring. Anesthesia was maintained using sevoflurane (end-tidal concentration adjusted to achieve a target BIS of 40–60) in combination with a continuous remifentanil infusion. In the remimazolam group (Group R), anesthesia was induced and maintained with remimazolam (Byfavo^®^, Hana Pharm, Seoul, Republic of Korea; induction, 6–12 mg/kg/h; maintenance, 1–2 mg/kg/h), titrated to achieve a BIS of 40–60 and supplemented with a continuous remifentanil infusion. Anesthetic dosing was not protocolized beyond the ranges specified in this section; within these ranges, hypnotic and opioid doses were titrated at the discretion of the attending anesthesiologist. Although the target BIS range was 40–60 in both groups, deeper anesthesia and neuromuscular blockade were frequently maintained at the surgeons’ request to facilitate fine dissection during the robotic and reconstructive procedures. BIS values were recorded in the anesthesia record at 10 min intervals.

In both groups, remifentanil (Ultian^®^, Hanlim Pharm, Seoul, Republic of Korea) was administered via continuous infusion (0.05–0.3 µg/kg/min) and titrated at the discretion of the attending anesthesiologist to maintain hemodynamic stability. Neuromuscular blockade was monitored using train-of-four (TOF) stimulation. Rocuronium (Esmeron^®^, MSD, Rahway, NJ, USA) 50 mg was administered as an intubating dose, and tracheal intubation was performed after confirmation of a TOF count of zero, followed by a continuous rocuronium infusion titrated to maintain a TOF count of two during surgery. A balanced crystalloid solution was infused at approximately 5 mL/kg/h, and 500 mL of 6% hydroxyethyl starch 130/0.4 in a balanced electrolyte solution (Volulyte^®^, Fresenius Kabi, Bad Homburg, Germany) was administered to all patients because of the prolonged operative duration.

During surgery, continuous subcutaneous CO_2_ insufflation was maintained at a target pressure of 6–8 mmHg to establish the working space. The patient remained in the supine position throughout the mastectomy phase. Following completion of the mastectomy, the operating table was adjusted to a semi-sitting position with ipsilateral arm elevation for implant pocket creation and prosthesis placement. The operative position remained unchanged until the completion of surgery.

At the end of surgery, all patients in Group R received flumazenil (Flunil^®^, Bukwang Pharmaceutical, Seoul, Republic of Korea) 0.2 mg intravenously to reverse remimazolam-induced sedation, with additional 0.1 mg increments administered as needed until adequate awakening was achieved. Intraoperative sufentanil (Sufental^®^, BC World Pharm, Seongnam, Republic of Korea) was administered at a dose of 0.05–0.1 µg/kg for analgesia toward the end of surgery in both groups. Neuromuscular blockade was reversed with sugammadex (Bridion^®^, MSD, Rahway, NJ, USA) or neostigmine–glycopyrrolate, as appropriate.

For postoperative nausea and vomiting (PONV) prophylaxis, palonosetron 0.075 mg (Aloxi^®^, Helsinn Healthcare SA, Lugano, Switzerland) was administered intravenously before the end of surgery in all patients. Postoperative analgesia was provided via IV patient-controlled analgesia with fentanyl, with the regimen individualized by the attending anesthesiologist according to body weight.

### 2.4. Hemodynamic Monitoring and Vasopressor Management

Standard monitoring included three-lead electrocardiography, pulse oximetry, invasive arterial blood pressure measurement, and BIS monitoring. In patients with available hemodynamic data, pulse pressure variation (PPV) was derived from the invasive arterial waveform, and cardiac output (CO) and cardiac index (CI) were measured using the FloTrac system (Edwards Lifesciences, Irvine, CA, USA) at the discretion of the attending anesthesiologist. Values were recorded at 5 min intervals throughout the procedure. Blood pressure values were extracted automatically from the electronic anesthesia record from anesthesia induction to the end of anesthesia and verified against the original anesthesia charts; invasive arterial pressure was used whenever it was being recorded, and noninvasive measurements were used only when invasive values were unavailable. Physiologically implausible values (e.g., readings recorded before arterial line connection) were excluded, and all hypotension-related outcomes were derived using the same rules for all patients. Duration below MAP 65 mmHg was estimated from 5 min MAP recordings, and the time-weighted average (TWA) was calculated as the area under the 65-mmHg threshold divided by valid MAP observation time.

Intraoperative hypotension was defined as MAP < 65 mmHg. Norepinephrine initiation was not strictly protocolized. A continuous norepinephrine infusion was used as the first-line vasopressor at the discretion of the attending anesthesiologist, either in response to MAP < 65 mmHg or preemptively while MAP remained ≥ 65 mmHg, based on the trajectory of blood pressure—for example, when MAP was declining rapidly and hypotension was anticipated. Consequently, norepinephrine consumption reflects clinician-driven management as well as patient physiology. In cases of acute hypotension requiring immediate intervention, ephedrine or phenylephrine was administered as an intermittent bolus before or in conjunction with the norepinephrine infusion. Because no validated conversion method was established for this study, the administration frequencies and cumulative doses of ephedrine and phenylephrine were reported separately, without conversion to norepinephrine equivalents. The vasopressor-free interval was defined as the time from anesthesia induction to the first administration of any vasopressor (norepinephrine infusion or ephedrine or phenylephrine bolus); patients who received no vasopressor were censored at the end of anesthesia. Episodes of more severe hypotension, defined as MAP < 60 mmHg, were also recorded and reported as supplementary hemodynamic outcomes.

### 2.5. Data Collection

The following data were extracted from electronic medical records: patient demographics (age, height, weight, and body mass index [BMI]), ASA physical status classification, comorbidities (hypertension, diabetes mellitus, chronic obstructive pulmonary disease, asthma, and coronary artery disease), smoking status, history of PONV, menopausal status, and prior chemotherapy or radiotherapy. The preoperative hemoglobin level was recorded as the sole laboratory variable.

Tumor and surgical characteristics included laterality, multifocality, histological subtype, tumor stage, lymph node status, cancer stage, type of lymph node surgery, reconstruction type, surgical margin status, tumor size, tumor weight, and biomarker status (estrogen receptor, progesterone receptor, human epidermal growth factor receptor 2, and Ki-67 index). The Ki-67 proliferation index was dichotomized as low (<20%) or high (≥20%) according to the institutional pathology reporting convention. For one patient in each group with no residual tumor on final pathology, tumor size was recorded as 0 cm.

Intraoperative data included the total doses of remimazolam, remifentanil, rocuronium, and sugammadex; average end-tidal sevoflurane concentration; anesthesia time, operative time, and robotic console time (all converted from hours and minutes to minutes); intraoperative sufentanil consumption (recorded in micrograms and converted to IV morphine equivalents using a conservative conversion factor of 0.7 [i.e., 1 µg sufentanil = 0.7 mg IV morphine, based on equianalgesic tables in which 14 µg sufentanil ≈ 10 mg IV morphine]) [16,17]; and vasopressor-related variables, including total norepinephrine consumption and intermittent ephedrine or phenylephrine use. Hemodynamic parameters (MAP, HR, CO, CI, and PPV) were extracted at eight predefined time points: baseline (pre-induction), post-induction, start of the surgical procedure, robot docking (robot-in), robot undocking (robot-out), start of plastic surgery, sitting position, and end of surgery.

Postoperative data included numeric rating scale (NRS) pain scores, opioid consumption (recorded as IV morphine equivalents), and the incidence of nausea and vomiting at three time points: in the post-anesthesia care unit (PACU) and at 24 and 48 h postoperatively. Data on rescue antiemetic use, PACU duration, length of hospital stay, flap-related adverse events, and the need for reoperation were also collected. Reconstructive complications were classified as severe (requiring an intervention such as unplanned reoperation or debridement; flap necrosis requiring surgical intervention; implant exposure or removal; or wound infection requiring parenteral antibiotics) or mild (transient and self-resolving without intervention, including localized erythema, discoloration, or swelling resolving within 1–2 days). Only severe events were subjected to formal comparative analysis, given the inherent limitations of retrospective data collection for minor findings.

### 2.6. Outcome Measures

The primary outcome was total intraoperative norepinephrine consumption (µg). Secondary outcomes included (1) vasopressor infusion duration (minutes); (2) vasopressor-free interval from induction (minutes); (3) incidence of intraoperative hypotension, defined as MAP < 65 mmHg; (4) hemodynamic profiles (MAP, HR, CO, CI, and PPV) across the eight predefined time points; (5) postoperative pain scores and opioid consumption in the PACU and at 24 and 48 h postoperatively; (6) incidence of PONV; and (7) severe reconstructive complications and length of hospital stay. The administration frequency and cumulative dose of each intermittent vasopressor (ephedrine and phenylephrine) were reported as supplementary descriptive outcomes. As post hoc sensitivity outcomes, norepinephrine dose was standardized by body weight and anesthesia duration (µg/kg/min), and hypotension burden was assessed using the estimated duration and TWA below MAP 65 mmHg.

### 2.7. Statistical Analysis

Continuous variables are presented as the mean ± standard deviation or median (interquartile range [IQR]) according to their distribution (Shapiro–Wilk test) and were compared using the independent-samples t-test or Mann–Whitney U test, as appropriate. Categorical variables are presented as n (%) and were compared using the chi-square test or Fisher’s exact test. Baseline balance was described using absolute standardized mean differences (SMDs).

Hemodynamic variables measured at multiple time points (MAP, HR, CO, CI, and PPV) were analyzed using a linear mixed-effects model with group, time, and group × time interaction as fixed effects and a patient-level random intercept to account for repeated measurements within individuals. This approach accommodates the missing CO, CI, and PPV values in patients without advanced hemodynamic monitoring.

A multivariable linear regression model for norepinephrine consumption included age, BMI, and anesthesia time as covariates, which were selected on clinical grounds because of their potential associations with intraoperative vasopressor requirements; given the limited sample size, covariate adjustment was deliberately parsimonious. Hypertension was not included as a covariate because only six patients had hypertension, which would have yielded an unstable estimate. Model assumptions were assessed using residual diagnostics, variance inflation factors, and Cook’s distance; because the residuals deviated from normality, a percentile-bootstrap 95% confidence interval (5000 resamples) was also calculated for the group coefficient. Sensitivity analyses repeated the model with ln(norepinephrine consumption + 1) as the dependent variable, with tumor size as an additional covariate (post hoc), and after excluding the observation with the largest Cook’s distance.

The vasopressor-free interval was compared between groups using Kaplan–Meier curves and the log-rank test, with censoring as defined in Section 2.4. All consecutively eligible patients were included, and no a priori sample size calculation was performed. Secondary and multivariable analyses were considered exploratory; no adjustment for multiple comparisons was applied to secondary analyses. A two-sided *p*-value < 0.05 was considered statistically significant. All analyses were performed using R version 4.4 (R Foundation for Statistical Computing, Vienna, Austria).

## 3. Results

**Figure 1:**
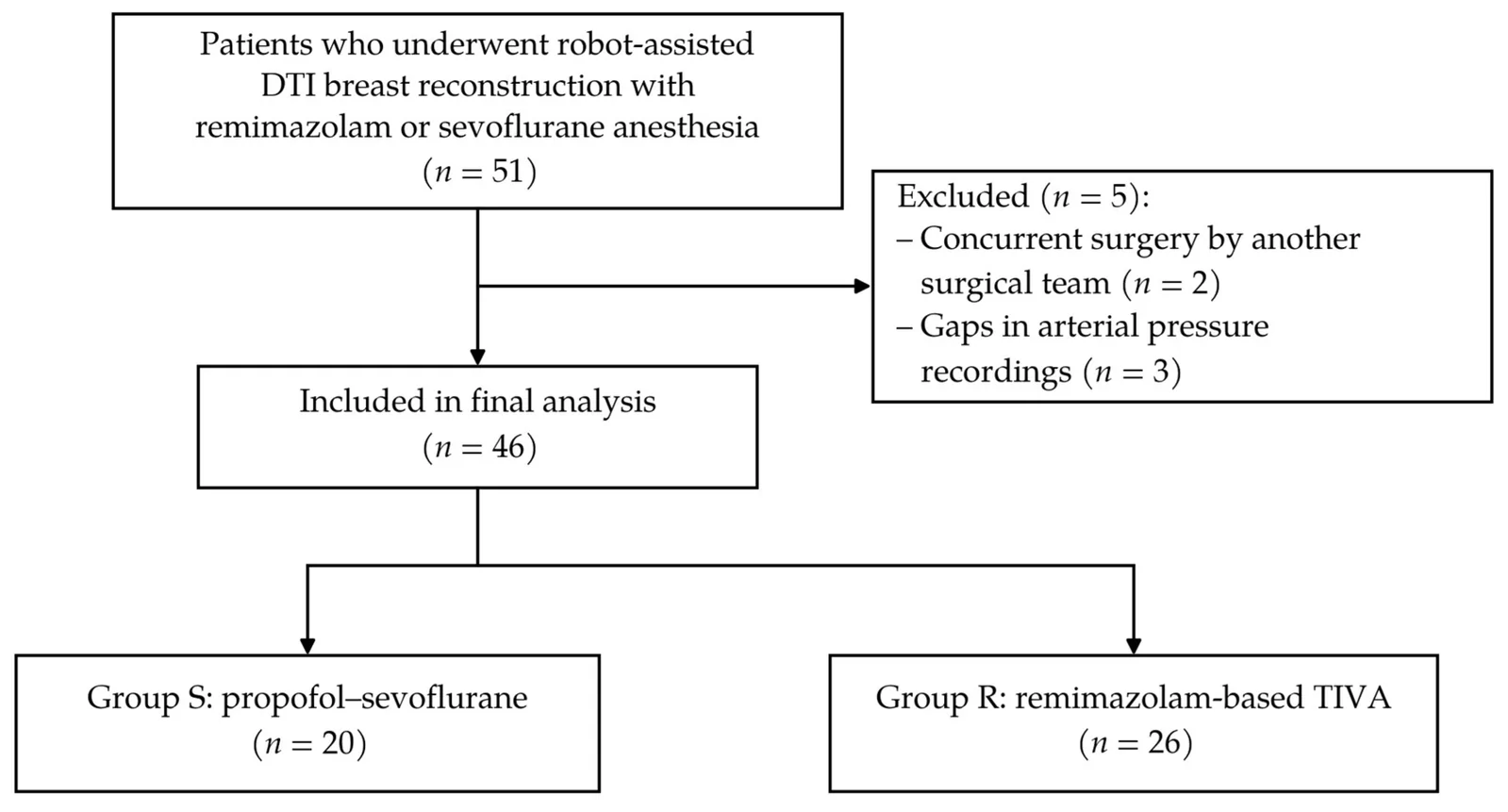
Flow diagram of patient selection. DTI, direct-to-implant; TIVA, total intravenous anesthesia.

Between November 2021 and September 2023, 51 consecutive patients underwent robot-assisted nipple-sparing mastectomy with immediate DTI breast reconstruction. Five patients were excluded: two underwent concurrent surgery performed by another surgical team, and three had substantial gaps in intraoperative arterial blood pressure recordings because of technical recording errors and were excluded at the screening stage, before data extraction. Isolated missing measurements alone did not result in exclusion. The patient-selection flow diagram is shown in Figure 1. No patient was excluded because of an intraoperative change in the surgical plan or postoperative ICU admission. In the present cohort, the first robot-assisted DTI case managed with remimazolam-based TIVA occurred in November 2021, and the first case managed with propofol–sevoflurane anesthesia occurred in June 2022. From June 2022 onward, both strategies were used concurrently, although remimazolam-based TIVA was used more frequently in 2023 (Supplementary Table S3). Surgical dates did not differ significantly between the groups (Mann–Whitney U test, *p* = 0.34); however, this does not exclude temporal confounding.

The baseline demographic, clinical, and tumor characteristics are presented in Table 1. No statistically significant differences were detected in age, BMI, ASA physical status, smoking status, hypertension, history of PONV, menopausal status, preoperative hemoglobin level, or preoperative chemotherapy (Table 1); however, non-significant *p*-values were not interpreted as evidence of baseline comparability. Diabetes mellitus, chronic obstructive pulmonary disease, asthma, and coronary artery disease were recorded but not tabulated because none of the patients had these comorbidities. Tumor characteristics, including laterality, multifocality, T stage, lymph node status, cancer stage, type of lymph node surgery, and biomarker profiles (estrogen receptor, progesterone receptor, human epidermal growth factor receptor 2, and Ki-67 index), did not differ significantly between the groups. Tumor size was greater in Group S than in Group R (3.20 ± 1.62 vs. 2.07 ± 1.11 cm; *p* = 0.008; SMD = 0.813). Anesthesia, operative, and robotic console times did not differ significantly between the groups (Table 2).

The intraoperative anesthetic data are summarized in Table 2. In Group S, propofol was administered at a median dose of 70 (IQR, 60–70) mg for co-induction, with sevoflurane maintained at a mean end-tidal concentration of 1.68 ± 0.10 vol%. In Group R, the median total remimazolam dose was 329.2 (IQR, 297.1–413.3) mg. Remifentanil consumption was significantly higher in Group R (median, 1520 [IQR, 1238–1767] vs. 742 [IQR, 641–846] µg; *p* < 0.001). Rocuronium consumption was also significantly higher in Group R (median, 200 [IQR, 150–200] vs. 150 [IQR, 150–150] mg; *p* = 0.002). Remifentanil and rocuronium were titrated at the discretion of the attending anesthesiologist within the same institutional ranges and targets in both groups; their higher consumption in Group R was not mandated by a group-specific dosing protocol. Intraoperative sufentanil consumption and total fluid input did not differ significantly between the groups (Table 2). Intermittent bolus vasopressor use was uncommon and did not differ significantly between the groups. Ephedrine was administered to 3 patients (15.0%) in Group S and 2 patients (7.7%) in Group R (*p* = 0.640), whereas no patients in Group S and 3 patients (11.5%) in Group R received phenylephrine (*p* = 0.246).

**Figure 2:**
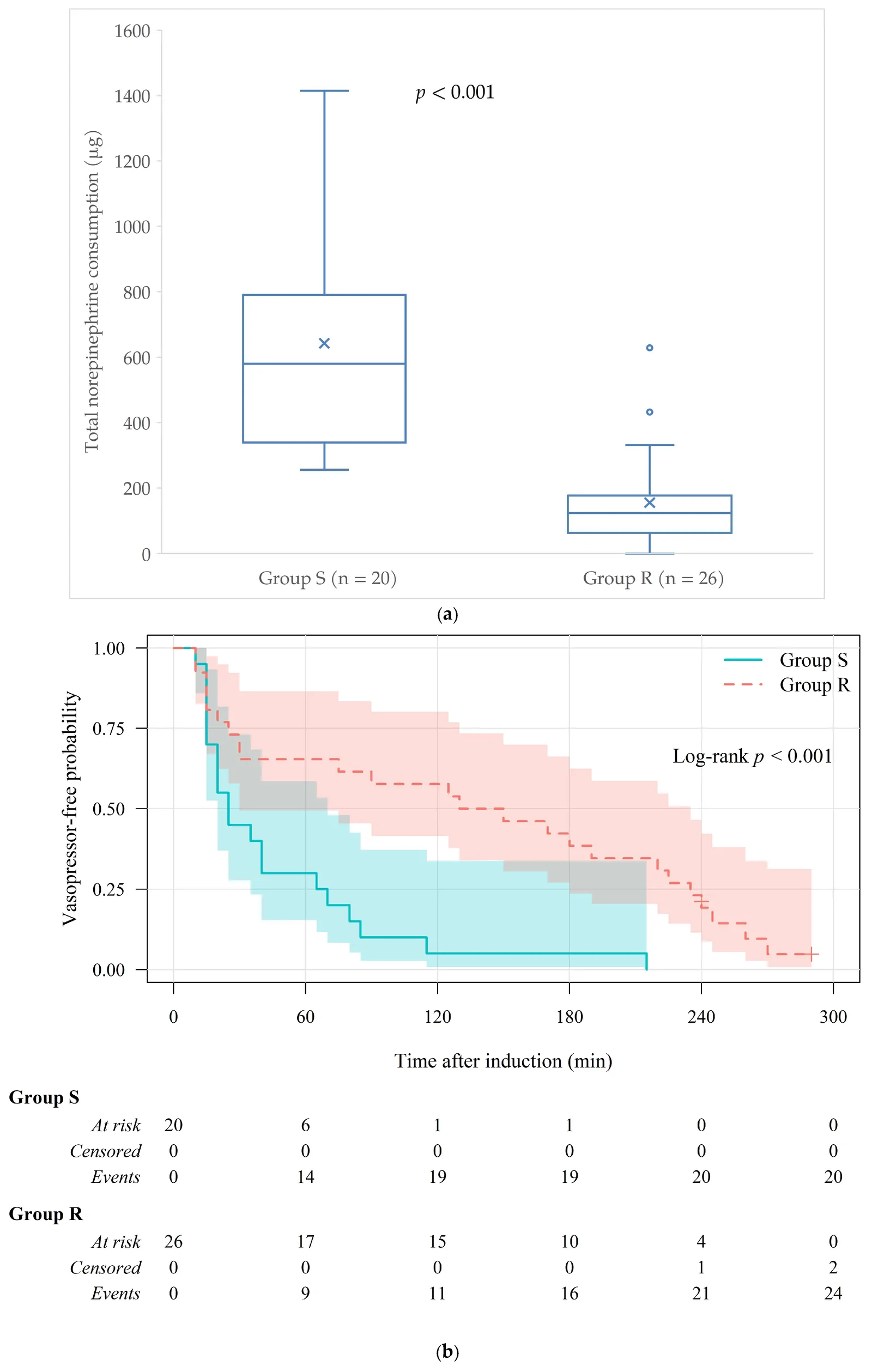
Intraoperative vasopressor requirements. (**a**) Total intraoperative norepinephrine consumption. The box represents the interquartile range, the horizontal line indicates the median, and the × symbol indicates the mean. Whiskers extend to values within 1.5 times the interquartile range; circles indicate outliers. Between-group differences were assessed using the Mann–Whitney U test (*p* < 0.001). (**b**) Kaplan–Meier curve of the vasopressor-free interval from induction to the first administration of any vasopressor. Patients who did not receive any vasopressor were censored at the end of anesthesia. Crosses (+) indicate censored observations. Between-group differences were assessed using the log-rank test (*p* < 0.001).

Total intraoperative norepinephrine consumption was significantly lower in Group R than in Group S (median, 123.2 [IQR, 62.4–176.4] vs. 580.0 [IQR, 338.8–790.4] µg; *p* < 0.001) (Figure 2a). The weight- and anesthesia-time-normalized norepinephrine dose was also significantly lower in Group R than in Group S (median, 0.00700 [IQR, 0.00377–0.0126] vs. 0.0370 [IQR, 0.0286–0.0570] µg/kg/min; *p* < 0.001).

Group S, propofol–sevoflurane anesthesia; Group R, remimazolam-based total intravenous anesthesia.

After adjustment for age, BMI, and anesthesia time, Group R was associated with lower norepinephrine consumption (*B* = −515.4 µg; 95% confidence interval, −661.3 to −369.4; *p* < 0.001; R^2^ = 0.561) (Table 3). Because the residuals deviated from normality (Shapiro–Wilk *p* = 0.025) and the maximum Cook’s distance was 0.380, the robustness of this estimate was examined further (Supplementary Table S2). The bootstrap 95% CI for the group coefficient was −674.7 to −356.1 µg. The observation with the largest Cook’s distance was a Group S patient with the highest norepinephrine consumption (1414.4 µg); excluding this patient attenuated the coefficient to −466.5 µg (95% CI, −599.2 to −333.8; *p* < 0.001), but its direction and statistical significance were preserved.

In an exploratory sensitivity analysis that additionally adjusted for tumor size, Group R remained associated with lower norepinephrine consumption than Group S (*B* = −517.4 µg; *p* < 0.001). The association also remained significant in a sensitivity analysis using log-transformed norepinephrine consumption, with Group R showing an exponentiated group coefficient of 0.14 relative to Group S (95% CI, 0.07–0.28; *p* < 0.001) (Supplementary Table S2). Vasopressor infusion duration was significantly shorter in Group R than in Group S (67.7 ± 40.0 vs. 176.3 ± 66.4 min; *p* < 0.001). Kaplan–Meier analysis showed that the vasopressor-free interval from induction was significantly longer in Group R than in Group S (median, 140 [95% CI, 30–235] vs. 25 [95% CI, 20–70] min; log-rank *p* < 0.001) (Table 2 and Figure 2b). Intraoperative hypotension (MAP < 65 mmHg) occurred in 80.0% of patients in Group S and 46.2% of those in Group R (*p* = 0.032). Neither the estimated duration of MAP < 65 mmHg nor the TWA MAP deficit below 65 mmHg differed significantly between the groups. The estimated duration was 0 (IQR, 0–10.0) min in Group R versus 7.5 (IQR, 5.0–16.3) min in Group S (*p* = 0.077), and the TWA MAP deficit was 0 (IQR, 0–0.138) versus 0.153 (IQR, 0.0248–0.343) mmHg, respectively (*p* = 0.056) (Table 2).

**Figure 3:**
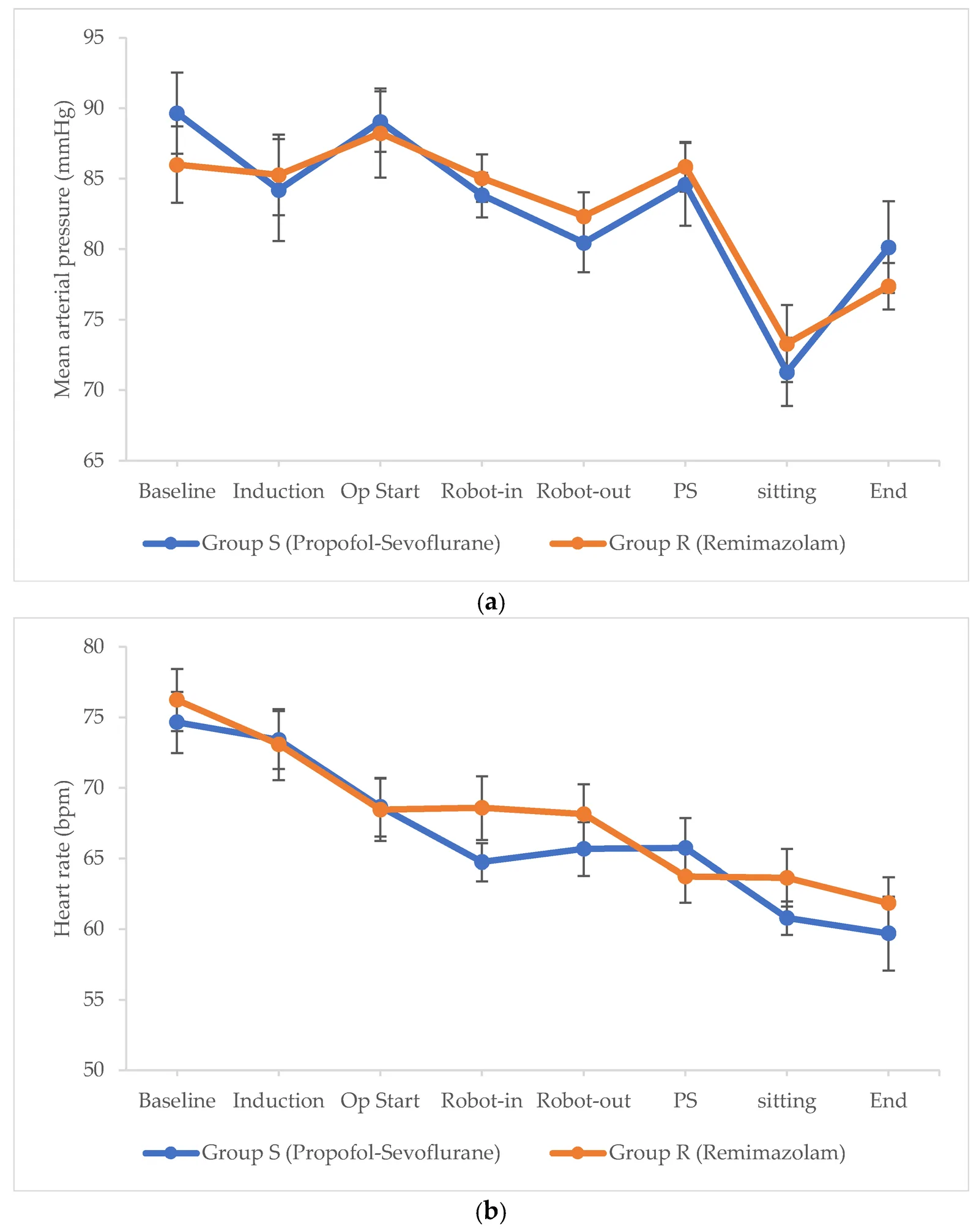
Hemodynamic profiles across eight predefined intraoperative time points. Serial measurements of (**a**) mean arterial pressure and (**b**) heart rate in Group S (propofol–sevoflurane, blue) and Group R (remimazolam, orange) at eight predefined time points. Data are presented as the mean ± standard error. Linear mixed-effects models showed a significant effect of time for both variables (both *p* < 0.001) but no significant group effect or group × time interaction for either variable (all *p* > 0.5). Mean arterial pressure: group, *p* = 0.942; time, *p* < 0.001; group × time interaction, *p* = 0.833. Heart rate: group, *p* = 0.517; time, *p* < 0.001; group × time interaction, *p* = 0.596. Op Start, start of surgery; Robot-in, robot docking; Robot-out, robot undocking; PS, start of plastic surgery; Sitting, placement in the semi-sitting position; End, end of surgery.

Across the eight time points, mean MAP ranged from 71.3 to 89.7 mmHg in Group S and from 73.3 to 88.2 mmHg in Group R, whereas mean HR ranged from 59.7 to 74.7 bpm and from 61.9 to 76.2 bpm, respectively (Figure 3). Linear mixed-effects models for MAP and HR demonstrated a significant effect of time (both *p* < 0.001) but no significant group effect or group × time interaction (MAP: group, *p* = 0.942; interaction, *p* = 0.833; HR: group, *p* = 0.517; interaction, *p* = 0.596) (Figure 3). Similarly, CO and CI, available for a subset of patients with FloTrac monitoring (Group S, *n* = 14–15; Group R, *n* = 21–23), showed no significant group effects or group × time interactions (CO: group, *p* = 0.909; interaction, *p* = 0.289; CI: group, *p* = 0.946; interaction, *p* = 0.259). PPV, available for a larger subset of patients with arterial monitoring (Group S, *n* = 18–19; Group R, *n* = 23–25), also showed no significant group effect or group × time interaction (group, *p* = 0.454; interaction, *p* = 0.872) (Supplementary Figure S1). BIS values were lower in Group S than in Group R (group effect, *p* < 0.001; group × time interaction, *p* < 0.001), with mean BIS values ranging from 29.3 to 35.3 in Group S and from 40.9 to 43.8 in Group R.

The postoperative outcomes are presented in Table 4. Pain scores did not differ significantly between the groups at any time point. PACU opioid consumption was significantly higher in Group R than in Group S in the unadjusted comparison (median, 0 [IQR, 0–3.3] vs. 0 [IQR, 0–0] mg morphine equivalents; *p* = 0.018), although this finding should be interpreted cautiously given the multiple postoperative comparisons. Opioid consumption at 24 and 48 h, incidence of PONV, rescue antiemetic use, PACU duration, and length of hospital stay did not differ significantly between the groups.

Severe reconstructive complications were infrequent in both groups: three patients (15.0%) in Group S and one patient (3.8%) in Group R underwent revision surgery or wound debridement (*p* = 0.303). No implant loss occurred in either group. Mild transient findings, including localized erythema, breast or nipple discoloration, and mild swelling, were documented in four patients in Group R (15.4%) and none in Group S; all findings resolved spontaneously within 1–2 days without intervention and did not delay hospital discharge. Oncologic treatment variables are summarized in Supplementary Table S1.

## 4. Discussion

In this retrospective cohort study comparing two anesthetic strategies in patients undergoing robot-assisted immediate DTI breast reconstruction, the remimazolam-based TIVA strategy was associated with significantly lower intraoperative norepinephrine consumption (median, 123.2 vs. 580.0 µg; *p* < 0.001), shorter vasopressor infusion duration (67.7 ± 40.0 vs. 176.3 ± 66.4 min; *p* < 0.001), and a lower observed incidence of intraoperative hypotension (MAP < 65 mmHg in 46.2% vs. 80.0%; *p* = 0.032) than the propofol–sevoflurane strategy. Serial MAP and heart rate did not differ between groups (Figure 3); however, because norepinephrine was titrated to maintain MAP, similar MAP values in the context of greater vasopressor use cannot be interpreted as evidence of comparable or superior intrinsic hemodynamic stability. Moreover, because norepinephrine was often started before MAP fell below 65 mmHg, recorded hypotension incidence and burden are themselves treatment-dependent outcomes. The absence of a significant difference in the estimated duration and TWA below MAP 65 mmHg, despite a several-fold difference in norepinephrine exposure, is therefore consistent with vasopressor titration preventing hypotension rather than with equivalent intrinsic hemodynamic stability. Accordingly, the present data support a difference in recorded vasopressor exposure but do not establish a reduction in cumulative hypotension burden.

These findings are of potential clinical relevance in the reconstructive context. Unlike conventional two-stage reconstruction with tissue expanders, DTI reconstruction places a permanent implant at the time of mastectomy, making adequate skin-flap perfusion an immediate prerequisite for a successful outcome [5,6,18]. The combination of semi-sitting positioning and CO_2_ insufflation during robot-assisted breast procedures may further increase the hemodynamic burden by reducing venous return, predisposing patients to intraoperative hypotension and to increased vasopressor requirements [7]. Against this backdrop, anesthetic agents that independently reduce systemic vascular resistance or depress myocardial contractility may impose an additional burden in an already hemodynamically vulnerable setting [8,9,10].

In this study, the propofol–sevoflurane strategy was associated with earlier vasopressor administration and a higher observed incidence of MAP < 65 mmHg. Kaplan–Meier analysis demonstrated a significantly shorter vasopressor-free interval in Group S than in Group R (median, 25 vs. 140 min; log-rank *p* < 0.001), and the incidence of intraoperative MAP < 65 mmHg was 80.0% in Group S compared with 46.2% in Group R, with a similar, although not statistically significant, pattern for more severe hypotension (MAP < 60 mmHg; 50.0% vs. 19.2%; *p* = 0.055; Supplementary Table S1). These findings may be consistent with the known dose-dependent reductions in systemic vascular resistance and CO produced by sevoflurane [10], although systemic vascular resistance was not measured in the present study. Conversely, the lower vasopressor requirement with the remimazolam-based strategy may be consistent with the cardiovascular profile of remimazolam, an ultrashort-acting benzodiazepine metabolized by tissue esterases that exerts minimal negative inotropic effects and produces less vasodilation at clinical doses than either propofol or volatile anesthetics [11,12,19]. This finding is also consistent with evidence demonstrating lower rates of hypotension and reduced vasopressor requirements with remimazolam than with propofol- or volatile-based regimens [13,14,15,20,21]. The availability of flumazenil as a specific antagonist allows predictable and readily reversible sedation [22].

However, these observations should be interpreted as reflecting the overall anesthetic strategy rather than the isolated pharmacological effects of any single agent. Although mastectomy flap perfusion was not directly assessed, intraoperative hypotension has been reported as a factor associated with skin-flap ischemia during implant-based reconstruction [23]. This consideration is particularly relevant when a definitive implant is placed during the index operation, as in DTI reconstruction. The clinical relevance of the present findings for mastectomy skin-flap perfusion is uncertain, and the direction of any effect remains unclear. Mastectomy skin flaps are random-pattern flaps that depend on the subdermal plexus, and cutaneous vessels are rich in α1-adrenergic receptors; greater norepinephrine exposure could therefore theoretically compromise flap microcirculation. Conversely, in free-flap reconstruction, perioperative vasopressor use has not been associated with increased flap failure, although flap complications appeared more frequent in breast reconstruction [24], and norepinephrine preserved flap blood flow better than phenylephrine in free transverse rectus abdominis myocutaneous flap breast reconstruction [25], suggesting that maintenance of perfusion pressure may matter more than vasopressor exposure per se. Whether these findings extend to mastectomy skin flaps is unknown, and recent prospective data indicate that surgical and patient factors, such as preservation of the second intercostal perforator and prior radiotherapy, are major determinants of flap necrosis [26]. Because flap perfusion was not measured and reconstructive complications were few, no inference regarding flap outcomes can be drawn from the present data. Prospective studies incorporating quantitative indocyanine green angiography are needed to determine whether the anesthetic strategy influences flap perfusion independently of surgical factors.

Two co-interventions inherent to the anesthetic strategies warrant explicit consideration. Remifentanil consumption was approximately twofold higher in Group R. Because remifentanil was titrated within the same range in both groups at the discretion of the attending anesthesiologist, this difference was not mandated by a group-specific dosing protocol and may partly reflect differences inherent to the anesthetic strategies. Unlike sevoflurane, which provides spinally mediated immobility and some antinociception, remimazolam has no intrinsic analgesic properties; thus, antinociception and immobility during remimazolam-based TIVA depend more heavily on the opioid component. Similarly, the higher rocuronium consumption in Group R is consistent with the potentiation of neuromuscular blockade by volatile anesthetics. Because higher remifentanil exposure would be expected to reduce sympathetic tone and blood pressure, this co-intervention is unlikely to account for the lower vasopressor requirement in Group R; nevertheless, the observed difference in vasopressor requirements cannot be attributed to the hypnotic component alone. PACU opioid consumption was also significantly higher in Group R (median, 0 [IQR, 0–3.3] vs. 0 [IQR, 0–0] mg morphine equivalents; *p* = 0.018), although the absolute values were small; this finding may partly reflect the rapid offset of remifentanil analgesia at emergence [27,28,29], although the underlying mechanisms were not directly evaluated. With respect to reconstructive outcomes, severe complications were infrequent in both groups (Group S, 3/20 vs. Group R, 1/26; *p* = 0.303), and this comparison was not sufficiently powered to detect a clinically meaningful difference. Mild transient findings documented in four patients in Group R and none in Group S resolved without intervention. Given the small number of events, these observations are descriptive.

This study had several limitations. First, its retrospective, single-center design and nonrandomized treatment allocation, which was based on attending anesthesiologist preference and drug availability, preclude causal inference, and selection bias and confounding by indication cannot be excluded. Data were obtained from anesthesia records that were not designed for research purposes; MAP was recorded at 5 min intervals, which may have missed brief hypotensive episodes, and BIS values were recorded at 10 min intervals, from which only values at the eight predefined time points were extracted. Although both strategies were used concurrently from June 2022 onward, remimazolam was used more frequently in the later period, and temporal changes in practice cannot be excluded. Furthermore, attending anesthesiologist and surgeon identities were not included in the analytic dataset. Consequently, residual confounding related to clinician-specific practice patterns, procedural learning curves, and other unmeasured factors cannot be excluded.

Second, the comparison was between two bundled anesthetic strategies rather than isolated hypnotic agents. Differences in remifentanil administration and neuromuscular blocker use may have contributed to the observed differences. Achieved BIS values differed substantially between the strategies (Group S, 29.3–35.3; Group R, 40.9–43.8) despite an identical target range. This difference likely reflects the institutional practice of maintaining deep anesthesia and neuromuscular blockade at the surgeons’ request for fine robotic and reconstructive dissection. In Group S, this was readily achieved by increasing the sevoflurane concentration, which also provides spinally mediated immobility and potentiates neuromuscular blockade, whereas in Group R, the same surgical conditions appear to have been achieved predominantly through greater remifentanil and rocuronium administration. In addition, agent-specific electroencephalographic characteristics, including increased beta-band activity reported during remimazolam sedation [30], may keep BIS values relatively high and limit direct comparisons of BIS values across anesthetic agents. Nevertheless, deeper effective anesthesia in Group S cannot be excluded as a contributor to greater norepinephrine exposure, and residual confounding by anesthetic depth remains a limitation.

In addition, norepinephrine initiation and titration were not fully protocolized. Preemptive administration and the limited temporal resolution of the 5 min MAP recordings may partly explain why some patients received norepinephrine without a recorded MAP < 65 mmHg. Because norepinephrine was frequently initiated preemptively on the basis of blood pressure trends, its consumption reflects both the patient’s hemodynamic trajectory and clinician judgment. Clinicians were not blinded to the anesthetic strategy, and expectations regarding agent-specific hemodynamic effects may have influenced the timing of initiation. Postoperative opioid consumption may also have been influenced by individualized patient-controlled analgesia regimens.

Third, several aspects of outcome assessment were limited. CO, CI, and PPV were available only for subsets of patients because advanced hemodynamic monitoring was applied at the discretion of the attending anesthesiologist; these subset analyses should therefore be interpreted as supportive rather than confirmatory. Mastectomy flap perfusion was not directly assessed, and postoperative follow-up was limited to the in-hospital period. Because some skin-flap, wound, and implant-related complications may become apparent after discharge, reconstructive event rates may have been underestimated. The sample size was small and was determined by the number of eligible cases rather than by an a priori power calculation. Although the difference in the primary outcome was large, the estimates are imprecise; the multivariable model could accommodate only a limited number of covariates; and the study was underpowered for secondary outcomes—particularly reconstructive complications, for which a clinically meaningful difference could not be excluded. Moreover, the single-center setting limits generalizability. No established patient-important threshold for intraoperative norepinephrine exposure exists in this surgical setting; therefore, the magnitude of the observed difference should not be interpreted as evidence of clinical benefit.

Despite these limitations, to our knowledge, this study is the first direct comparison of remimazolam-based TIVA and propofol–sevoflurane anesthetic strategies in robot-assisted DTI breast reconstruction and provides preliminary effect-size estimates to inform the design of adequately powered prospective trials.

## 5. Conclusions

In this retrospective, single-center cohort of patients undergoing robot-assisted immediate DTI breast reconstruction, a remimazolam-based TIVA strategy was associated with lower recorded intraoperative norepinephrine use than a propofol–sevoflurane strategy. No significant between-group differences in serial MAP or HR were detected. The clinical implications of these associations, including any effects on skin-flap perfusion or reconstructive outcomes, require prospective evaluation using standardized protocols.

## Figures and Tables

**Table 1:** Baseline patient demographic and tumor characteristics.

	Group S (*n* = 20)	Group R (*n* = 26)	*p*-Value	SMD
**Demographics**				
Age (years)	47.8 ± 8.9	46.4 ± 9.9	0.641	0.140
Height (cm)	159.0 ± 4.8	160.5 ± 4.9	0.302	0.311
Weight (kg)	54.3 (51.8–58.8) ^†^	55.1 (52.4–61.5) ^†^	0.595 ^‡^	0.250
BMI (kg/m^2^)	22.0 ± 2.3	22.3 ± 2.9	0.682	0.125
ASA physical status			0.184 ^§^	0.550
I	2 (10.0)	0 (0)		
II	18 (90.0)	25 (96.2)		
III	0 (0)	1 (3.8)		
Smoking status			0.364 ^§^	0.414
Never	16 (80.0)	24 (92.3)		
Former	3 (15.0)	2 (7.7)		
Current	1 (5.0)	0 (0)		
Hypertension, n (%)	4 (20.0)	2 (7.7)	0.380 ^§^	0.362
History of PONV, n (%)	1 (5.0)	4 (15.4)	0.369 ^§^	0.348
Menopause, ¶ n (%)	3 (18.8)	5 (21.7)	>0.999 ^§^	0.074
Preoperative hemoglobin (g/dL)	13.0 (12.0–13.6) ^†^	12.8 (12.2–13.5) ^†^	0.842 ^‡^	0.059
Preoperative chemotherapy, n (%)	1 (5.0)	5 (19.2)	0.212 ^§^	0.447
**Tumor characteristics**				
Laterality (left), n (%)	7 (35.0)	9 (34.6)	>0.999 ^§^	0.008
Multifocality, n (%)	9 (45.0)	8 (30.8)	0.369 ^§^	0.297
Tumor size (cm)	3.20 ± 1.62	2.07 ± 1.11	0.008	0.813
Specimen weight (g)	385 (252–494) ^†^	305 (233–378) ^†^	0.266 ^‡^	0.298
T stage, n (%)			0.634 ^§^	0.429
Tis	8 (40.0)	9 (34.6)		
T1	8 (40.0)	10 (38.5)		
T2	3 (15.0)	7 (26.9)		
T3	1 (5.0)	0 (0)		
Lymph node status, n (%)			0.889 ^§^	0.460
N0	17 (85.0)	22 (84.6)		
N1	3 (15.0)	2 (7.7)		
N2	0 (0)	1 (3.8)		
N3	0 (0)	1 (3.8)		
Cancer stage, n (%)			0.936 ^§^	0.249
0	8 (40.0)	9 (34.6)		
I	8 (40.0)	9 (34.6)		
II	3 (15.0)	6 (23.1)		
III	1 (5.0)	2 (7.7)		
Lymph node surgery, n (%)			0.405 ^§^	0.450
SLNB	18 (90.0)	19 (73.1)		
ALND	1 (5.0)	3 (11.5)		
Both	1 (5.0)	4 (15.4)		
ER positive, n (%)	16 (80.0)	20 (76.9)	>0.999 ^§^	0.075
PR positive, n (%)	13 (65.0)	14 (53.8)	0.551 ^§^	0.229
HER2 positive, n (%)	7 (35.0)	9 (34.6)	>0.999 ^§^	0.008
High Ki-67 index, n (%)	4 (20.0)	13 (50.0)	0.064 ^§^	0.663

Values are presented as the mean ± SD, median (interquartile range) ^†^, or n (%). ^‡^ Mann–Whitney U test. ^§^ Fisher’s exact test. ¶ Data were available for 39 patients (16 in Group S and 23 in Group R). SMDs are presented as absolute values and were calculated from the underlying data. SMD, standardized mean difference. Group S, propofol–sevoflurane anesthesia; Group R, remimazolam-based anesthesia. BMI, body mass index; ASA, American Society of Anesthesiologists; PONV, postoperative nausea and vomiting; SLNB, sentinel lymph node biopsy; ALND, axillary lymph node dissection; ER, estrogen receptor; PR, progesterone receptor; HER2, human epidermal growth factor receptor 2.

**Table 2:** Intraoperative anesthetic, hemodynamic, and fluid-balance data.

	Group S (*n* = 20)	Group R (*n* = 26)	*p*-Value
**Anesthetic** **data**			
Propofol induction dose (mg)	70 (60–70) ^†^	—	*—*
End-tidal sevoflurane concentration (vol%)	1.68 ± 0.10	—	*—*
Total remimazolam dose (mg)	—	329.2 (297.1–413.3) ^†^	*—*
Total remifentanil dose (µg)	742 (641–846) ^†^	1520 (1238–1767) ^†^	<0.001 ^‡^
Total rocuronium dose (mg)	150 (150–150) ^†^	200 (150–200) ^†^	0.002 ^‡^
Sufentanil, IV morphine equivalents (mg)	3.2 (2.8–3.5) ^†^	3.2 (2.8–3.5) ^†^	0.863 ^‡^
**Operative times**			
Anesthesia time (min)	272.0 ± 39.9	285.6 ± 43.7	0.284
Operative time (min)	228.4 ± 38.9	242.1 ± 36.5	0.206
Robotic console time (min)	40 (32.5–47.5) ^†^	40 (34–60) ^†^	0.547 ^‡^
**Vasopressor consumption**			
Total norepinephrine consumption (µg)	580.0 (338.8–790.4) ^†^	123.2 (62.4–176.4) ^†^	<0.001 ^‡^
Weight- and anesthesia-time-normalized norepinephrine dose (µg/kg/min)	0.0370 (0.0286–0.0570) ^†^	0.00700 (0.00377–0.0126) ^†^	<0.001 ^‡^
Vasopressor infusion duration (min)	176.3 ± 66.4	67.7 ± 40.0	<0.001
Vasopressor-free interval from induction (min)	25 (20–70) ^‖^	140 (30–235) ^‖^	<0.001 ^‖^
Any episode of mean arterial pressure < 65 mmHg, n (%)	16 (80.0)	12 (46.2)	0.032 ^§^
Estimated duration of MAP < 65 mmHg (min)	7.5 (5.0–16.3) ^†^	0 (0–10.0) ^†^	0.077 ^‡^
TWA below MAP 65 mmHg (mmHg)	0.153 (0.0248–0.343) ^†^	0 (0–0.138) ^†^	0.056 ^‡^
Lowest mean arterial pressure (mmHg)	59.5 (54.0–64.0) ^†^	65.0 (61.0–67.0) ^†^	0.048 ^‡^
**Fluid balance**			
Total fluid input (mL)	1827.5 ± 365.8	1926.9 ± 451.3	0.427
Urine output (mL)	560 (500–840) ^†^	290 (200–390) ^†^	0.001 ^‡^
Estimated blood loss (mL)	30 (20–50) ^†^	50 (20–90) ^†^	0.098 ^‡^

Values are presented as the mean ± standard deviation, median (interquartile range) ^†^, or n (%). ^‡^ Mann–Whitney U test. ^§^ Fisher’s exact test. ^‖^ Log-rank test; values are Kaplan–Meier median estimates with 95% confidence intervals. Patients who did not receive any vasopressor were censored at the end of anesthesia. Group S, propofol–sevoflurane anesthesia; Group R, remimazolam-based anesthesia. IV, intravenous; MAP, mean arterial pressure; TWA, time-weighted average.

**Table 3:** Multivariable linear regression analysis of total intraoperative norepinephrine consumption.

Variable	*B* (95% CI)	SE	*t*	*p*-Value	VIF
Intercept	−67.0 (−916.5 to 782.5)	422.1	−0.16	0.875	—
Group (R vs. S)	−515.4 (−661.3 to −369.4)	72.3	−7.13	<0.001	1.03
Age (years)	−0.2 (−7.9 to 7.4)	3.8	−0.06	0.951	1.01
BMI (kg/m^2^)	10.4 (−17.7 to 38.6)	14.0	0.75	0.459	1.02
Anesthesia time (min)	1.8 (0.1–3.6)	0.9	2.08	0.043	1.05

Model: R^2^ = 0.561; adjusted R^2^ = 0.518; F(4, 41) = 13.1; *p* < 0.001. The dependent variable was total intraoperative norepinephrine consumption (µg). *B*, unstandardized regression coefficient; CI, confidence interval; SE, standard error; VIF, variance inflation factor; BMI, body mass index.

**Table 4:** Postoperative outcomes.

	Group S (*n* = 20)	Group R (*n* = 26)	*p*-Value
Pain scores (NRS, 0–10)			
PACU	3 (3–4) ^†^	3 (3–4) ^†^	0.344 ^‡^
24 h	3 (3–4) ^†^	3 (2–6) ^†^	0.566 ^‡^
48 h	2 (2–2) ^†^	2 (2–3) ^†^	0.308 ^‡^
Opioid consumption (IV morphine equivalents, mg)			
PACU	0 (0–0) ^†^	0 (0–3.3) ^†^	0.018 ^‡^
24 h	0 (0–1.5) ^†^	0 (0–1.5) ^†^	0.951 ^‡^
48 h	0 (0–2.2) ^†^	0 (0–2.2) ^†^	0.547 ^‡^
Postoperative nausea and vomiting, n (%)			
PACU nausea	4 (20.0)	3 (11.5)	0.682 ^§^
PACU vomiting	2 (10.0)	0 (0)	0.184 ^§^
24 h nausea	8 (40.0)	13 (50.0)	0.561 ^§^
24 h vomiting	0 (0)	1 (3.8)	>0.999 ^§^
48 h nausea	3 (15.0)	6 (23.1)	0.711 ^§^
Rescue antiemetic use within 48 h, n (%)	17 (85.0)	22 (84.6)	>0.999 ^§^
Recovery			
PACU duration (min)	40 (30–45) ^†^	40 (30–50) ^†^	0.689 ^‡^
Length of hospital stay (days)	7 (7–7) ^†^	7 (7–8) ^†^	0.174 ^‡^
Surgical outcomes			
Mild transient findings, n (%)	0	4 (15.4)	Descriptive only
Severe reconstructive complications, n (%)	3 (15.0)	1 (3.8)	0.303 ^§^

Values are presented as the mean ± standard deviation, median (interquartile range) ^†^, or n (%). ^‡^ Mann–Whitney U test. ^§^ Fisher’s exact test. Group S, propofol–sevoflurane anesthesia; Group R, remimazolam-based anesthesia. NRS, numeric rating scale; PACU, post-anesthesia care unit; IV, intravenous. No vomiting was observed in either group at 48 h.

## Data Availability

The original contributions presented in this study are included in the article/Supplementary Material. Further inquiries can be directed to the corresponding author.

## Supplementary Materials

The following supporting information can be downloaded at: https://www.mdpi.com/article/10.3390/medicina62101907/s1. Figure S1: Additional hemodynamic profiles across intraoperative time points; Table S1: Supplementary hemodynamic and oncologic treatment variables; Table S2: Sensitivity analyses for total intraoperative norepinephrine consumption; Table S3: Temporal distribution of cases by group.

## Abbreviations

The following abbreviations are used in this manuscript:

DTI	Direct-to-implant
CO_2_	Carbon dioxide
TIVA	Total intravenous anesthesia
IV	Intravenous
IRB	Institutional Review Board
ASA	American Society of Anesthesiologists
BIS	Bispectral index
TOF	Train-of-four
ICU	Intensive care unit
PPV	Pulse pressure variation
CO	Cardiac output
CI	Cardiac index
HR	Heart rate
MAP	Mean arterial pressure
TWA	Time-weighted average
BMI	Body mass index
PONV	Postoperative nausea and vomiting
NRS	Numeric rating scale
PACU	Post-anesthesia care unit
SMD	Standardized mean differences
SD	Standard deviation
IQR	Interquartile range
VIF	Variance inflation factor
