# Supplementary material for: Hemodynamic Outcomes and Vasopressor Requirements with Remimazolam-Based Total Intravenous Anesthesia Versus Propofol–Sevoflurane Anesthesia in Robot-Assisted Direct-to-Implant Breast Reconstruction
Source: Medicina (Kaunas). 2026 Oct 2;62(10):1907. doi: 10.3390/medicina62101907 (PMC13707097; doi:10.3390/medicina62101907)
Supplement: Supplementary file 1 [file medicina-62-01907-s001.zip › medicina-4594840-supplementary.pdf]

Supplementary Materials

(**a**)

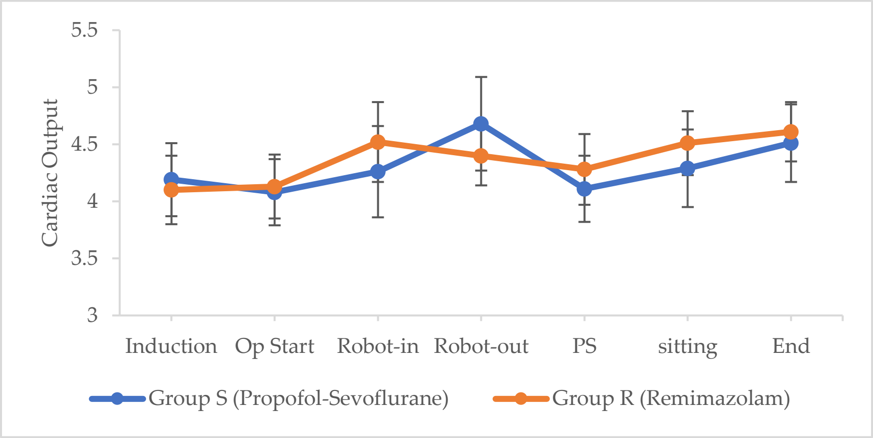

(**b**)

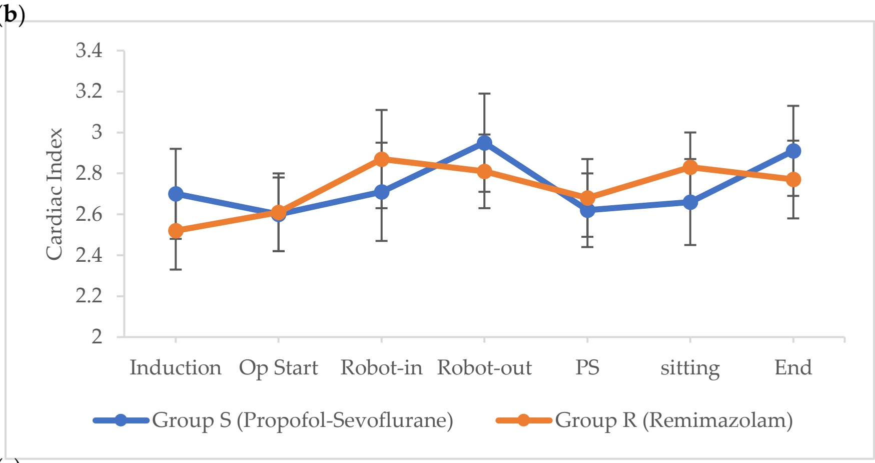

(**c**)

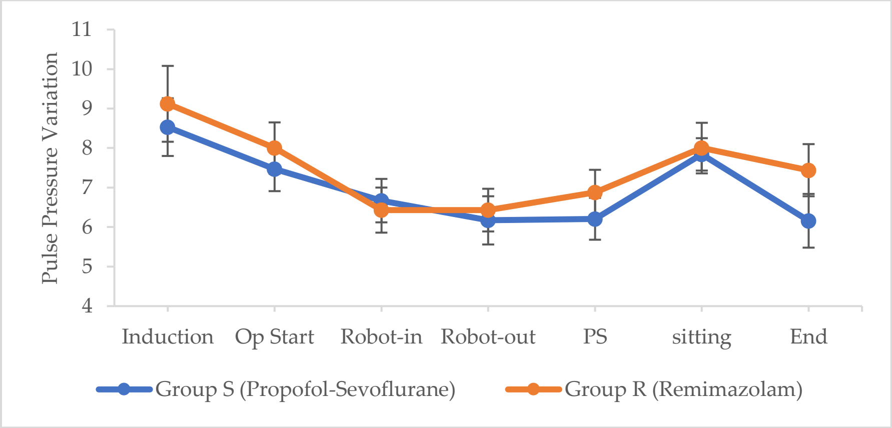

Figure S1. Additional hemodynamic profiles across intraoperative time points.
Serial measurements of (**a**) cardiac output, (**b**) cardiac index, and (**c**) pulse pressure variation in groups S (propofol–sevoflurane, blue) and R (remimazolam, red) at seven predefined time points (baseline excluded, as these parameters were available only after induction). Cardiac output and cardiac index were available for a subset of patients who underwent FloTrac monitoring (Group S, n = 14–15; Group R, n = 21–23). The pulse pressure variation was derived from the invasive arterial waveform and was available in a larger subset (Group S, n = 18–19; Group R, n = 23–25). Data are presented as mean ± standard error of the mean. PS: Plastic surgery start. Linear mixed-effects models showed no significant group effect or group × time interactions for any variable.

(A) Cardiac output: group $p = 0.909$; time $p < 0.001$; group × time interaction $p = 0.289$. (B) Cardiac index: group $p = 0.946$; time $p = 0.010$; group × time interaction $p = 0.259$. (C) Pulse pressure variation: group $p = 0.454$; time $p < 0.001$; group × time interaction $p = 0.872$.

Table S1. Supplementary hemodynamic and oncologic treatment variables

	**Group S (*n* = 20)**	**Group R (*n* = 26)**	***p*-value**
**Intraoperative hemodynamic data**			
Mean arterial pressure < 60 mmHg, any episode, n (%)	10 (50.0)	5 (19.2)	0.055
Ephedrine use, n (%)	3 (15.0)	2 (7.7)	0.640
Phenylephrine use, n (%)	0	3 (11.5)	0.246
**Oncologic treatment variables**			
Adjuvant chemotherapy	2 (10.0)	8 (30.8)	0.150
Adjuvant radiotherapy	3 (15.0)	7 (26.9)	0.476
Hormonal therapy	15 (75.0)	18 (69.2)	0.749

Values are presented as n (%). Group S, propofol–sevoflurane anesthesia; Group R, remimazolam-based anesthesia. All *p*-values were calculated using Fisher's exact test.

Table S2. Sensitivity analyses for total intraoperative norepinephrine consumption

Model	Group effect, Group R vs Group S	*p*-value
Primary adjusted model	B = −515.4 μg (95% CI, −661.3 to −369.4)	< 0.001
Primary model, percentile bootstrap (5000 resamples)	B = −515.4 μg (95% CI, −674.7 to −356.1)	—
Excluding the observation with the largest Cook's distance (0.380)	B = −466.5 μg (95% CI, −599.2 to −333.8)	< 0.001
Tumor size-adjusted model	B = −517.4 μg (95% CI, −678.8 to −355.9)	< 0.001
Log-transformed model	adjusted ratio = 0.14 (95% CI, 0.07 to 0.28)	< 0.001

All models included 46 patients. The primary model was adjusted for age, body mass index, and duration of anesthesia. The tumor size-adjusted model also included tumor size. In the log-transformed model, norepinephrine consumption was transformed to ln(norepinephrine consumption + 1) because two patients had no norepinephrine exposure. The group effect was presented as an exponentiated adjusted ratio. Diagnostics of the primary model: Shapiro–Wilk test of residuals, W = 0.943, p = 0.025; variance inflation factors, 1.01–1.05; maximum Cook's distance, 0.380 (mean, 0.030). The observation with the largest Cook's distance was a Group S patient with the highest norepinephrine consumption (1414.4 μg; DFBETA for the group coefficient, −48.9 μg). The influence analysis was a post hoc sensitivity analysis. B, unstandardized regression coefficient; CI, confidence interval.

Table S3. Temporal distribution of cases by group

Group	2021 Q4	2022 Q1	2022 Q2	2022 Q3	2022 Q4	2023 Q1	2023 Q2	2023 Q3	Total
Group S	0	0	4	4	5	5	0	2	20
Group R	1	0	4	3	3	8	2	5	26
Total	1	0	8	7	8	13	2	7	46

Values are numbers of patients by quarter of surgery. 2021 Q4 includes November–December 2021 only. The first case managed with remimazolam-based total intravenous anesthesia occurred in November 2021 and the first case managed with propofol–sevoflurane anesthesia in June 2022. Surgical dates did not differ significantly between the groups (Mann–Whitney U test, p = 0.34). Group S, propofol–sevoflurane anesthesia; Group R, remimazolam-based anesthesia.
